# Thiazide and thiazide-like diuretics in nephrolithiasis

**DOI:** 10.1590/2175-8239-JBN-2019-0148

**Published:** 2020-11-11

**Authors:** Tamara da Silva Cunha, Samirah Abreu Gomes, Ita Pfeferman Heilberg

**Affiliations:** 1 Universidade Federal de São Paulo, Escola Paulista de Medicina, Disciplina de Nefrologia, São Paulo, Brasil.; 2 Universidade Federal do Rio de Janeiro, Departamento de Nefrologia, Rio de Janeiro, Brasil.; 3 Faculdade de Medicina da Universidade de São Paulo, Laboratório de Nefrologia Celular, Genética e Molecular, Departamento de Clínica Médica, São Paulo, Brasil.

**Keywords:** Diuretics, Hydrochlorothiazide, Hypercalciuria, Indapamide, Kidney Stones, Urolithiasis., Diurético, Hidroclorotiazida, Hipercalciúria, Indapamida, Cálculos Renais, Urolitíase.

## Abstract

Thiazide and thiazide-like diuretics are widely used for the management of hypercalciuria among stone-forming patients. Although the effects of different thiazides should be relatively similar in terms of prevention of stone recurrence, their potency and side effects may differ. However, there is scarce data concerning the metabolic and bone effects of these agents among recurrent nephrolithiasis patients with hypercalciuria. The aim of this update article was to compare our experience in the use of thiazide and thiazide- like diuretics with that of the current literature, concerning their anticalciuric properties and consequent reduction of recurrent stone formation. Their impact on bone mass and potential side effects were also discussed.

## Resumo

Diuréticos tiazídicos e *tiazídicos-like* são amplamente usados para o tratamento da hipercalciúria em pacientes com formação de cálculos. Embora os efeitos dos diferentes tiazídicos devam ser relativamente semelhantes em termos de prevenção da recorrência do cálculo, sua potência e efeitos colaterais podem ser diferentes. No entanto, há poucos dados sobre os efeitos metabólicos e ósseos desses agentes em pacientes com nefrolitíase recorrente com hipercalciúria. O objetivo deste artigo de atualização foi comparar nossa experiência quanto ao uso de tiazídicos e *tiazídicos-like* com a publicada na literatura atual, no que diz respeito às suas propriedades anticalciúricas e consequente redução da formação de cálculos recorrentes. Discutimos também seu impacto na massa óssea e potenciais efeitos colaterais.

## Introduction

Kidney stone recurrence can be either symptomatic through multiple episodes of renal colic or manifested as new stone formation of previous stone growth. Idiopathic hypercalciuria represents the most frequent metabolic disorder found in stone formers, affecting approximately 50% of all patients^1^, and thiazide and thiazide-like diuretics are widely used for both treatment and prevention of recurrence. Besides lowering the total urinary calcium excretion due to augmented distal calcium reabsorption, increased proximal tubule reabsorption of sodium and calcium (driven by volume contraction) can provide further benefits because of the diminished delivery of calcium to the distal medullar interstitium. These effects, associated to reduced urinary pH and calcium phosphate supersaturation, may contribute to a potential decrease in Randall’s plaque formation^2^.

Although the effects of different thiazides should be relatively similar in terms of prevention of stone recurrence or treatment of hypertension, Reilly et al^3^ have stressed about evidence-based distinctions with respect to the use and dosage of hydrochlorothiazide (HCTZ) compared to the thiazide-like diuretics such as chlorthalidone (CTD) or indapamide (IDP). Their potency and side effects also differ, and IDP can be distinguished from HCTZ for not containing the benzothiadiazine core. The risk of hyponatremia, hypokalemia, hyperuricemia, and dyslipidemia seems to be lower with IDP, but this still remains controversial since the differences have not been proven^4^. 

A recent highly debated potential side effect seen after the long-term use of thiazide or thiazide-like diuretics is their association with a higher risk of skin cancer, especially cutaneous and lip squamous cell carcinoma, due to their photosensitizing properties^5^^-^ 14.

Osteopenia has been often recognized among kidney stone patients presenting with long lasting idiopathic hypercalciuria, but whether bone resorption plays a primary or a secondary role in the pathogenesis of hypercalciuria still remains unclear^15^. Thiazide and thiazide-like diuretics, by their ability to decrease urinary calcium excretion, could be particularly useful for ameliorating bone mass or even decreasing bone resorption in hypercalciuric patients with osteopenia^16^. In an experimental model of hypercalciuric rats with nephrolithiasis, the use of thiazides not only reduced urinary calcium excretion, but also ameliorated trabecular bone quality^17^. Previous studies conducted in the general population have shown protective effects of thiazides against hip fracture, that may disappear soon after its discontinuation^18^.

However, the majority of studies comparing different types of thiazides have focused on the effect of lowering blood pressure and/or their adverse effects in the hypertensive population. Therefore, there is scarce data concerning the metabolic and bone effects of different thiazides among recurrent nephrolithiasis patients with hypercalciuria.

The aim of this update article was to compile data about the use of thiazides and their potential side effects in hypercalciuric stone formers and also provide some personal experience of the authors in this field. 

## Discussion

### Metabolic and Clinical Aspects of Thiazide and Thiazide-Like Diuretics Use in Nephrolithiasis

In order to compare our experience in the use of thiazide and thiazide-like diuretics with that of the current literature regarding metabolic aspects and bone effects of these drugs, we performed a retrospective analysis of the medical records of recurrent kidney stone outpatients with idiopathic hypercalciuria followed up at the Universidade Federal de São Paulo (UNIFESP), who had previously been on a regular therapy with HCTZ (25 mg/day) or IDP (2.5 mg/day) for at least one year (*unpublished data*). Patients with evidence of heart, liver or endocrine diseases, postmenopausal females, and those with abnormal renal function were excluded, but not those with hypertension. For the purpose of the present analysis, we just selected patients who had available data concerning serum and urinary biochemistry before starting the use of these drugs and after around three months on treatment. Data regarding dual energy X-ray absorptiometry (DEXA) before and after 1 year on treatment were retrieved from a total of 28 patients (35±11 years old), 17 men and 11 women, with a mean BMI (body mass index) of 25.9±3.2 for IDP (n=11) and 27.9±3.6 kg/m^2^ for HCTZ (n=17) groups, respectively. The initial values of mean blood pressure did not differ significantly between the group taking HCTZ compared with the one taking IDP (99.2 ± 10.0 mmHg and 102.7 ± 14 mmHg, respectively), but the achieved values after three months were significantly lower for HCTZ versus IDP (88.5 ± 12.0 vs. 96.0 ± 13.0 mmHg, *p* <0.001). Regarding side effects, one patient reported both sexual dysfunction and dizziness with HCTZ, suggesting hypotension, and was lost to follow-up. No other severe side effect had been reported in the medical records during this short period of time. Urinary and blood results are shown in Table 1. There was a significant and similar reduction of urinary calcium after treatment with either HCTZ or IDP and a significant increase in mean urinary sodium only in those receiving IDP. Urinary uric acid and citrate did not differ between groups. The expected, comparable, and significant reduction of urinary calcium excretion observed after the first three months on treatment was in accordance with previous reports in stone formers^19^^,^ 20. In the present series, the employed dose of HCTZ (25 mg/d) was lower compared to previous studies which claimed that indapamide 2.5 mg/d corresponds to HCTZ 50 mg/d in terms of control of urinary calcium levels^21^^,^ 22. Importantly, as shown in Table 2, most of the randomized clinical trials (RCTs) conducted in the past employing thiazides focused on prevention of new stone formation as the main outcome, with a marked reduction on the risk of recurrence in most of them^23^^-^ 29 with the exception of three who conducted less than 2 years of follow-up^30^^-^ 32. Nevertheless, all but two of these studies (assessing trichlormethiazide and indapamide)^27^^,^ 28, had been designed for stone forming patients who were not selected on the basis of urinary calcium excretion and employed only high doses of thiazides (50 to 100 mg). Curiously, the stone incidence has been lowered even among normocalciuric patients^23^^-^ 26, what suggests that the less calcium in the urine the better, even when within the normal range. In summary, meaningful data concerning dose-response effects upon calciuria is still lacking. Nowadays, lower doses of thiazides are often employed aiming to reduce metabolic adverse effects, but their efficacy as such is not well established^3^^,^ 21 ^,^ 33. In order to clarify these unsolved questions, a 3-year prospective double-blind ongoing trial was initiated in 2017 with the purpose of assessing the efficacy of standard and low dose HCTZ in the recurrence prevention of calcium stone formation^34^.

**Table 1:** Serum and urinary parameters before (pre) and after (post) treatment

	Indapamide		Hydrochlorothiazide	
Serum	*Pre*	*Post*	*Pre*	*Post*
Calcium (mg/dL)	9.3 ± 0.5	9.6 ± 0.9	10.0 ± 0.1	10.0 ± 0.1
Potassium (mEq/L)	4.5 ± 0.3	4.0 ± 0.4*	4.3 ± 0.4	4.0 ± 0.3
Uric Acid (mg/dL)	5.3 ± 0.8	5.9 ± 1.8	4.6 ± 1.3	6.3 ± 2.6*
Cholesterol (mg/dL)	194 ± 45	200 ± 52	185 ± 34	200 ± 24
Triglycerides (mg/dL)	115 ± 68	117 ± 71	117 ± 99	116 ± 96
Glucose (mg/dL)	91 ± 12	95 ± 21	76 ± 9.3	83 ± 10.8
**Urine**				
Calcium (mg/kg/24h)	4.4 ± 0.9	3.0 ± 0.9*	5.0 ± 1.3	3.3 ± 0.7*
Sodium (mEq/24h)	223 ± 67	271 ± 77*	195 ± 90	221 ± 123
Uric Acid (mg/24h)	612 ± 181	716 ± 231	574 ± 405	512 ± 392
Citrate (mg/24h)	510 ± 370	470 ± 288	439 ± 187	461 ± 161

Data are reported as mean ± SD;

* p<0.05 (versus pre).

**Table 2:** Randomized clinical trials of thiazide and thiazide-like diuretics in stone formers

Author, year	Drug	Dose	Selection for Hypercalciuria	Number treated/placebo	RR for Recurrence	Followup (years)
Brocks, 1981	Bendroflumethiazide	2.5 mg TID	No	33/29	NS	1.6
Scholz, 1982	HCTZ	25 mg BID	No	25/26	NS	1
Mortensen, 1986	Bendroflumethiazide + KCl	2.5 mg TID	No	12/10	NS	2
Laerum, 1984	HCTZ	25 mg BID	No	25/25	0.39	3
Wilson, 1984	HCTZ	100 mg daily	No	23/21	0.48	2,8
Robertson, 1985	Bendroflumethiazide	2.5 mg TID	No	13/9	0.38	3-5
Ettinger, 1988	Chlorthalidone	25/50 mg	No	19 (25mg), 23 (50mg) /31 (placebo)	0.23	3
Ohkawa, 1992	Triclormetiazida	4 mg	Yes	82/93	0.42	2,1-2,2
Borghi, 1993	Indapamida	2.5 mg dia	Yes	43/14	0.21	3
Fernandes-Rodrigues, 2006	HCTZ	50 mg dia	No	50/50	0.56	3

HCTZ: hydrochlorothiazide; KCl: potassium chloride; BID: twice daily; TID: three times daily; RR: relative risk.

Notwithstanding the evidence of lower stone recurrence on thiazides compared to placebo, it is worth mentioning that most of the aforementioned studies had been conducted along the 1980s and 1990s. That said, the way to evaluate stone recurrence was through kidney/ureter/bladder (KUB) X-ray, intravenous pyelogram (IVP), or US every 6 to 12 months. Therefore, some of the flaws of such RCTs in the preceding era of computed tomography (CT) scans, relied on the difficulties regarding the identification of new calculi formation or growth of pre-existing ones in KUB X-ray alone as follow-up image. 

### Systemic and Metabolic Adverse Effects of Thiazide and Thiazide-Like Diuretics

Concerning metabolic side effects, a handful of clinical studies have disclosed them for stone formers using such drugs. In the present series, we observed that mean serum potassium was significantly lower versus baseline levels (although within the normal range) in the group taking IDP, which was not observed with HCTZ (Table 1). Although these findings may be ascribed to a higher baseline level of serum potassium in the former group, such data contrast with the one in the literature revealing less hypokalemia for IDP than HCTZ^3^. Conversely, the mean serum levels of uric acid were significantly higher for HCTZ than IDP, in agreement with other investigators^35^. Mean serum glucose and lipid levels did not differ after treatment nor between both drugs. This is in line with data from Singh et al^36^ which showed that stone formers who received thiazide therapy solely for kidney stone prophylaxis were not at increased risk for subsequent diabetes mellitus (DM). Lately, there have been several studies reporting associations with the long-term use of thiazide and thiazide-like diuretics with the risk of non-melanoma skin cancers^5^^-^ 14, as shown in Table 3. Association between use of HCTZ and malignant melanoma is less elucidated, but recent data have suggested increased risk (cumulative and dose-dependent) of nodular melanoma and lentigo melanoma^11^, what still warrants further investigation. A more recent meta-analysis of observational studies evaluating the association between the use of thiazides and the risk of skin cancers suggested that such use may be associated with an increased risk of skin cancers especially squamous cell carcinomas which reinforces the several studies mentioned above^37^. Of note, it is important to mention that many other antihypertensive drugs are also considered photosensitizers including not only thiazides, but e.g. loop diuretics, potassium-sparing agents, and alpha-2 receptor agonists, which may act as co-carcinogens under ultraviolet radiation (UVR) exposure^12^. Anyway, a note of caution concerning special care for sun exposure in thiazide users should be given. The short duration of follow-up in our retrospective analysis does not allow us any comment about such associations.

**Table 3:** Clinical studies about the use of thiazides and the risk of skin cancers

Author, year	Study design	Cancer type	Drug	Results
Jensen, 2008	Case-control	SCC	HCTZ + amiloride (>5 years)	IRR 1.79 (1.45-2.21)
Ruiter, 2010	Cohort	BCC	Thiazides	HR 1.0 (0.95-1.05)
Friedman, 2012	Case-control	SCC Lip cancer	HCTZ	OR 4.22 (2.82-6.31)
De Vries, 2012	Case-control	SCC	Thiazides	OR 1.66 (1.16-2.37)
Robinson, 2013	Case-control	SCC	Thiazides	OR 1.3 (0.7-2.4)
Schmidt, 2015	Case-control	SCC	Thiazides + potassium sparing agents	OR 2.68 (2.24-3.21)
Pottergard, 2017	Caso-controle	SCC CA lábio	HCTZ ≥ 25.000 mg	OR 3.9 (3.0-4.9)
			Thiazides	
Nardone, 2017	Coorte	BCC/SCC/MM	Thiazides	BCC: OR 2.11 1.60-2.79)
				SCC: OR 4.11 (2.66-6.35)
				MM: OR 1.82 (1.01-3.82)
Pedersen, 2018	Case-control	NMSC	HCTZ >50.000mg	BCC: OR 1.29 (1.23-1.35)
		(BCC/SCC)	(cumulative dose)	SCC: OR 3.98 (3.68-4.31)
Su, 2018	Cohort	SCC	Tiazídicos Thiazides	HR 1.09 (0.99-1.19)

SCC: squamous cell carcinoma; IRR: incidence rate ratio; OR: odds ratio (confidence interval); NMSC: nonmelanoma skin cancer; BCC: basal cell carcinoma; HR: hazard ratio; HCTZ: hydrochlorothiazide; MM: malignant melanoma.

### Bone Effects of Thiazide and Thiazide-Like Diuretics

**Figure 1:**
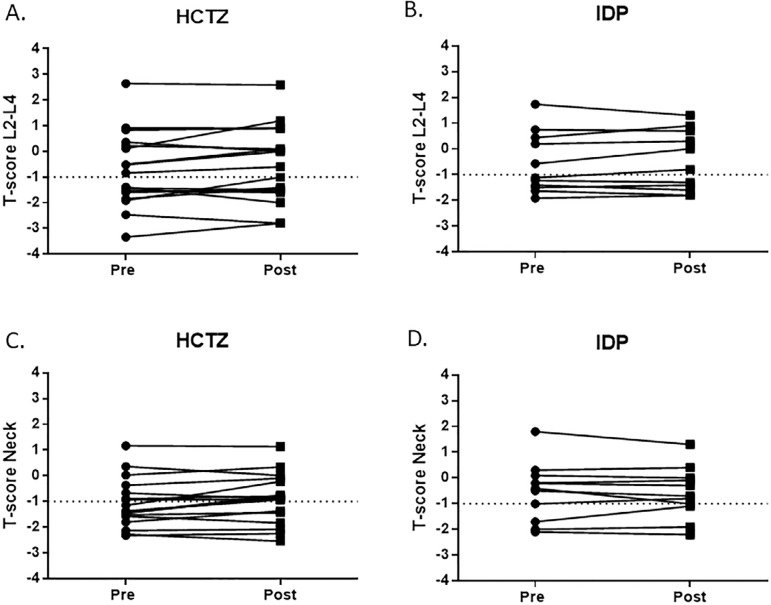
T-score individual values obtained by bone densitometry at lumbar spine (L2-L4) and neck sites in hypercalciuric stone formers before (pre) and after (post) one year on hydrochlorothiazide (HCTZ) or indapamide (IDP) therapy.

In the present study, the results of bone mineral density (BMD) obtained after one year on treatment showed that patients using HCTZ presented a slight, but not significant improvement (data not shown in tables) of the mean value of T-score in L_2_-L_4_ (-1.04 ± 0.36 versus -1.63 ± 0.28) and a significant increase in the femur neck (-1.22 ± 0.22 versus -1.55 ± 0.20, *p* < 0.05). The increases in mean T-score with IDP were less evident and not significant at both L_2_-L_4_ (-0.565 ± 1.2 versus -0.49 ± 1.2) and femur neck sites (-0.53 ? 1.1 versus -0.6 ? 1.0). However, there has been a wide variation of the individual BMD values pre- and post-treatment, as shown in Figure 1, with 11 out of 17 patients taking HCTZ presenting increases in T-score from 2.3 to 80.0% and 6 out of 11 taking IDP presenting increases in T-score from 5.0 to 42.0%. Both HCTZ and indapamide might decrease the risk of fractures by reducing urinary calcium excretion and enhancing calcium balance^38^^,^ 39. However, such effect of thiazide and thiazide-like diuretics on bone could also extend beyond its anti-calciuric actions since *in vitro* they may potentially stimulate osteoblastic bone formation enhancing osteocalcin production and expression of thiazide-sensitive sodium chloride co-transporter, which leads to osteoblastic proliferation^40^. Moreover, an inhibition of osteoclastic bone resorption was also observed and can be important to sustain the bone mass^16^. For all these reasons, thiazide therapy approach to improve bone mass and strength in hypercalciuric patients is recommended.

## Conclusion

Kidney stone treatment and prevention of recurrence are clinically important. Thiazide and thiazide-like diuretics have been widely used for the management of idiopathic hypercalciuria. However, there is a lack of RCT studies including hypercalciuric patients aiming to compare potency and side effects, employing different doses and classes of these agents, with a long-term follow-up. In our personal experience, HCTZ (25 mg daily) and indapamide (2.5 mg daily) were well tolerated as initial dose therapy and provided reduction of urinary calcium excretion with no severe side effects, with a promising protective effect on bone mass.
